# Genome-wide identification of differentially expressed genes under water deficit stress in upland cotton (*Gossypium hirsutum* L.)

**DOI:** 10.1186/1471-2229-12-90

**Published:** 2012-06-15

**Authors:** Wonkeun Park, Brian E Scheffler, Philip J Bauer, B Todd Campbell

**Affiliations:** 1 USDA-ARS, Coastal Plains Soil, Water and Plant Research Center, Florence, SC, USA; 2 USDA-ARS, MSA Genomics Laboratory, Stoneville, MS, USA

## Abstract

**Background:**

Cotton is the world’s primary fiber crop and is a major agricultural commodity in over 30 countries. Like many other global commodities, sustainable cotton production is challenged by restricted natural resources. In response to the anticipated increase of agricultural water demand, a major research direction involves developing crops that use less water or that use water more efficiently. In this study, our objective was to identify differentially expressed genes in response to water deficit stress in cotton. A global expression analysis using cDNA-Amplified Fragment Length Polymorphism was conducted to compare root and leaf gene expression profiles from a putative drought resistant cotton cultivar grown under water deficit stressed and well watered field conditions.

**Results:**

We identified a total of 519 differentially expressed transcript derived fragments. Of these, 147 transcript derived fragment sequences were functionally annotated according to their gene ontology. Nearly 70 percent of transcript derived fragments belonged to four major categories: 1) unclassified, 2) stress/defense, 3) metabolism, and 4) gene regulation. We found heat shock protein-related and reactive oxygen species-related transcript derived fragments to be among the major parts of functional pathways induced by water deficit stress. Also, twelve novel transcripts were identified as both water deficit responsive and cotton specific. A subset of differentially expressed transcript derived fragments was verified using reverse transcription-polymerase chain reaction. Differential expression analysis also identified five pairs of duplicated transcript derived fragments in which four pairs responded differentially between each of their two homologues under water deficit stress.

**Conclusions:**

In this study, we detected differentially expressed transcript derived fragments from water deficit stressed root and leaf tissues in tetraploid cotton and provided their gene ontology, functional/biological distribution, and possible roles of gene duplication. This discovery demonstrates complex mechanisms involved with polyploid cotton’s transcriptome response to naturally occurring field water deficit stress. The genes identified in this study will provide candidate targets to manipulate the water use characteristics of cotton at the molecular level.

## Background

Predicted world population growth will require more food, feed, and fiber production. Intermittent drought and shortages of water supply negatively impact crop productivity and are predicted to occur more frequently in future agricultural systems [1-3]. In future agricultural systems, an increase in crop productivity must also be accompanied by a reduced environmental impact of production on natural resources [4]. Therefore, to increase productivity while maintaining adequate water resources, it is necessary to understand the mechanisms plants use to cope with water deficit stress [5]. There have been two general efforts towards more sustainable and water use efficient agricultural production. One effort is represented by breeding and genetic engineering [1,6,7]. Another effort is focused on the improvement of agricultural practices such as tillage and irrigation systems [8-10]. To successfully pursue the breeding and genetic engineering approach, it is fundamental to understand plant responses to drought stress at the molecular, cellular, and genetics levels. This understanding is critical, because drought tolerance is a quantitative trait influenced by a combination of regulatory pathways [5,11].

Drought stress impacts a network of plant gene expression mechanisms that lead to the reprogramming of a variety of physiological and metabolic processes in accordance with stress response. Early studies primarily used model plant species to identify a wide spectrum of genes that are involved in different levels of metabolism, signal transduction, osmotic regulation, stress response, and gene regulation [12-14]. More recently, many plant gene expression profiling experiments, using a range of technical approaches, have been conducted on economically important crop plants to determine stress related expression signatures. For example, next generation sequencing technology (NGS) has been applied to provide global gene expression profiles of whole transcriptomes [15,16]. NGS expression studies are greatly facilitated by the availability of annotated genome sequence (reference or otherwise).

As an alternative to NGS, the cDNA-AFLP technique is a widely adopted gene expression profiling method. This is especially true for crop plants, such as cotton, that are awaiting whole genome- or whole transcriptome- sequence data. cDNA-AFLP profiling has proven to be a robust method to discover differentially expressed genes in a number of plant species [17-19]. With the advantage of no prerequisite reference genome sequence requirement, cDNA-AFLP has been used to understand regulation mechanisms under various biotic and abiotic stress conditions that influence patterns of global gene expression in diverse crop species [20-22].

Cotton, a warm climate crop of tremendous global economic importance, is known to contain intrinsic mechanisms that allow it to partially withstand water deficit- and heat-stresses by its deep and extensive root system and adaptive osmoregulation mechanisms [23,24]. However, as water deficit stress progressively continues, development and reproduction of cotton is severely affected, and the quality and yield of cotton fiber production is significantly reduced [25,26]. Therefore, for successful cotton fiber production, it is essential to understand proper management strategies between water demand, irrigation, and plant responses that are seasonally variable [27].

In cotton, there are several reports on the molecular regulatory mechanisms that orchestrate drought resistance using quantitative trait loci (QTL) mapping or by developing drought stress induced cDNA libraries [28-30]. In addition, microarray gene expression profiling has been used in cotton tissues exposed to variable water deficit stress in a controlled environment growth condition [31]. However, microarray gene expression profiling is limited to ESTs or genes present on the microarray chip and may not provide a complete representation of differentially expressed genes.

Although genome sequencing data of many economically important crop species are widely available as full or high quality draft formats [32], the progress of cotton genome sequencing has been slow. Genome sequencing of an ancestral diploid genome (D) is near completion [33,34]. Once complete, the D-genome sequence will be useful as a platform for more comprehensive information on cultivated cotton’s tetraploid genome [35].

In this study, our objective was to gain comprehensive insight into the water deficit stress-related gene expression profile in cotton grown under field conditions. To accomplish this, we used cDNA-AFLP to identify differentially expressed genes in the roots and leaves of a single cotton cultivar grown in a rain-fed, drought-prone field environment. From this large comparative analysis, fundamental experimental data illustrate complex mechanisms of gene expression in response to water deficit stress. This study should facilitate future efforts to improve the agricultural productivity of cotton and other crops under soil water deficit conditions.

## Results

### Collection of cDNA-AFLP data sets

**Figure 1:**
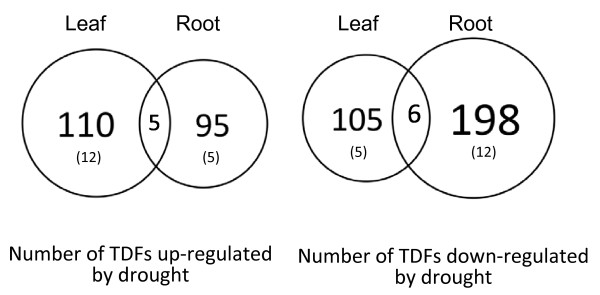
**Diagrammatic distribution of differentially expressed TDFs in leaf and root tissues.** Numbers in parentheses indicate reciprocally up- and down-regulated TDFs in two different tissues.

Figure 1 summarizes the distribution of TDFs in leaf and root tissues. In total, 519 differentially expressed fragments (13%) were detected among about 4,000 TDFs that demonstrated clear banding patterns produced by the LI-COR DNA analyzer. The TDFs ranged in fragment size from 50 to 700 bp. The remaining TDFs (87%) showed equal expression in the tissues examined regardless of the water deficit stress applied. From the total 519 differentially expressed TDFs, 210 (40.5%) showed increased expression and 309 TDFs (59.5%) showed decreased expression. Among 210 TDFs with increased expression, 115 TDFs were from leaf tissue and 100 TDFs from roots. Five TDFs showed increased expression in both leaf and root tissues. Among 309 TDFs with reduced expression upon water deficit stress, 111 TDFs were from leaf tissue and 204 were from root tissue. Six TDFs showed reduced expression in both tissues. In addition to the 519 differentially expressed TDFs, 17 additional TDFs showed different expression patterns between leaf and root tissues (shown in parentheses in Figure 1). Expression of 12 of the 17 TDFs was induced in the leaf while being reduced in root tissue. The remaining five TDFs showed reduced expression in the leaf and induced expression in the root.

Since the cDNA-AFLP procedure uses a two selective nucleotide extension at the 3’ terminus of each primer (*See methods*), there are 256 available primer combinations. In total, 64 primer combination covers one-quarter of the transcriptome of interest in tissues tested. Although the total gene number of the cotton transcriptome is not clear, it is estimated that the tetraploid cotton genome contains over 70,000 genes [36]. Therefore, we estimate that our expression data represents approximately 17,500 genes.

### Sequence analysis and functional annotations

**Figure 2:**
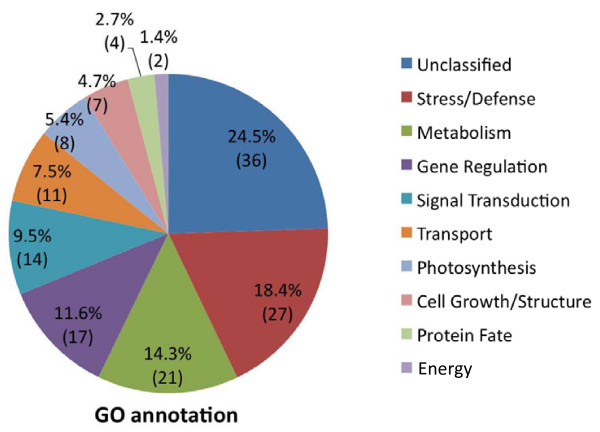
**Classification of differentially expressed TDFs.** Percentiles are indicated to show the ratio of 10 categories. Numbers in parentheses denote TDFs indentified in each category.

Of the 519 differentially expressed fragments, 366 bands were excised, recovered, cloned, and sequenced. Three or four colonies from each excised band were bi-directionally sequenced, and a total of 1,440 clones were analyzed for sequence identity. Finally, 147 independent TDFs were verified by sequence analysis after evaluating the sequence products with expected size, the existence of two selective nucleotide extension, and clone reproducibility (Additional file 1, GenBank accession numbers JK512212- JK512358). Sizes of the fragments ranged from 177 to 680 bp with the average length of 307 bp. Using criteria in the Gene Ontology annotation tool (http://www.geneontology.org/) [37], the functional annotation of TDFs was listed in ten categories (Figure 2). The majority of TDFs (68.8%) grouped into four categories; 1) metabolism, 2) stress/defense mechanism, 3) gene regulation (transcription, translation, etc), and 4) unclassified. Twenty-five TDFs functioning in signal transduction (9.5%) and transport (7.5%) were also detected. Twenty-one TDFs (14.3%) were assigned across four additional categories that included photosynthesis (5.4%), cell growth/cellular structure (4.7%), protein fate (2.7%), and energy (1.4%) (Figure 2 and Additional file 1). Among 36 unclassified TDFs, 16 belonged to conserved protein-coding genes, eight had sequence similarity to proteins of unknown function, and 12 did not show any sequence similarity to known protein sequences. One TDF (06E08) did not match any known protein or EST sequences in the NCBI database (Additional file 1). A cDNA library constructed from drought stressed *G. arboreum*, (a diploid cotton) indicated that 21.5% of clones had blast homology search similarity to unknown genes [29]. Similarly, our data showed 24.5% of TDFs with similarity to unknown genes.

### Differentially expressed genes under water deficit stress

Tissue specificity of annotated proteins is described in Additional file 1. Annotated proteins in the stress/defense category covered 18.4% (27 TDFs) of the total TDFs. Ten of these 27 TDFs coded for proteins related to the production of reactive oxygen species (ROS). Examples of ROS-related proteins included glutathione *S*-transferase (GST), manganase superoxide dismutase (Mn-SOD)-like protein, glutathione peroxidase, mono-dehydroascorbate reductase, phosphoadenosine phosphosulfate reductase (Thioredoxin), aldo/keto reductase, 5’-adenylylsulfate reductase (Glutathione), and superoxide dismutase (SOD) copper chaperone. Six of the 27 TDFs were annotated as heat shock proteins (HSP). Examples of annotated HSP included chloroplast HSP70.1-like proteins, HSP70, DnaJ HSP-like protein, and alpha-crystalline HSP. Also, of the 27 TDFs, two were annotated as alcohol dehydrogenase (ADH) enzymes, two as drought-induced proteins, and seven TDFs with relationships to other stress/defense responses.

**Figure 3:**
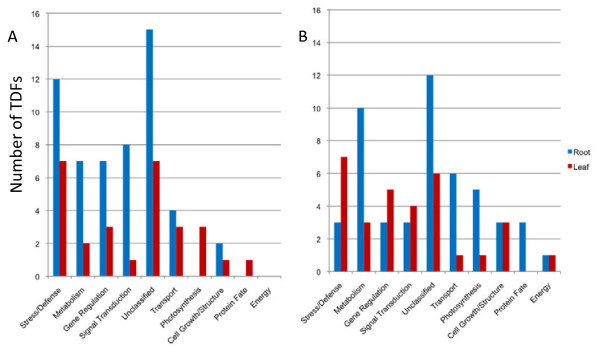
Comparison of TDF distribution in root and leaf tissues across functional categories: A) Up-regulated TDFs and B) Down-regulated TDFs.

Fifteen TDFs in the metabolism category had similarity to predicted proteins participating in the biosynthesis pathways of fatty acids, amino acids, and sugars. This suggests active changes in the composition of biologically important macromolecules during water deficit stress. Interestingly, in the metabolism category, the number of TDFs differentially expressed under water deficit stress was 3X higher in root tissue than in leaf tissue (Figure 3). This suggests more dynamic metabolomic changes in root than in leaf.

Among the 17 gene-regulation categorized TDFs, eight coded for transcription factors and seven for translation-related factors. The signal transduction category (14 TDFs, 9.5%) consisted of four kinase, four phosphatase, and four GTP binding factors. Two TDFs coding for aquaporin proteins (PIP1;3 and PIP1;12) were identified in leaf and root tissues. Five cell-wall biosynthesis-related genes were also identified (Additional file 1).

Figure 3 shows a comparison of up- (Figure 3A) and down-regulated (Figure 3B) TDFs in root and leaf tissues. Compared to leaf tissue, up-regulated TDFs were found more abundantly in the root across the five primary functional categories- unclassified, metabolism, stress/defense, signal transduction, and gene regulation. Down-regulation was more prominent in leaf tissue for stress/defense, signal transduction, and gene regulation. Down-regulation was more prominent in root tissue for metabolism, unclassified, and transport categories.

### Validation of expression pattern by RT-PCR

RT-PCR was performed using specific primers for TDFs proportionally representative of each functional category. Since our primary focus was on stress response, more TDFs from the stress/defense category were included for RT-PCR analysis. Table 1 summarizes TDFs selected for expression analysis. For each TDF, all RT-PCR reactions produced products of the expected size according to sequence information. Sixty percent showed the same expression pattern as that collected from the cDNA-AFLP data. Although amplified using gene-specific primers, it is possible that expression of duplicated TDF copies negatively affected RT-PCR amplification. Given the relatively short average size of TDFs and the complexity of the tetraploid cotton genome, more sequence information is necessary to obtain enough sequence data for gene specific primer design and amplification. Comparing TDF sequences and EST sequences with adequate sequence coverage (if available) would allow unique sequence regions to be determined for better primer design.

**Table 1:** **Summary of RT-PCR reactions performed for selected TDFs**^**a**^

**TDF ID**	**Description**	**Category**	**EST**	**cDNA-AFLP^b^**	**Sizì (bp)**	**RT-PCR^d^**
02 C11	Hypothetical	Unclassified	DT468917	up R	161	N
02 H11	Hypothetical		DW510601	up R	208	Y
02A12	Hypothetical		DW498397	up R	244	Y
06E08	no homology		na*	up R	186	Y
06B01	glycosyl transferase family 1 protein		ES813597	up L	225	N
01D09	nucleic acid binding protein/cold shock induced	Stress/Defense	ES820443	up R	268	Y
01B11	chloroplast heat shock protein 70.1		DT572113	up L up R	176	Y
02D03	glutathione S-transferase		DW495591	up L	320	N
02A05	Lea5-D/Drought induced		DW228770	up R	233	Y
02A09	manganese superoxide dismutase-like protein		DW226492	dn R	209	N
03E11	universal stress protein		DW484973	up R	350	N
04B09	Hsp70		DW486235	up R	275	Y
02 C06	phosphoethanolamine N-methyltransferase		DW238205	up R	505	N
07B12	phosphoadenosine phosphosulfate reductase family protein		EY197850	up R	273	Y
A04H12	Cu/Zn SOD		JG454758	up R	338	Y
03A07	beta-ketoacyl-ACP synthase II	Metabolism	DW238354	up R	333	Y
08 H05	ribose-5-phosphate isomerase		DT556372	up L	227	Y
08D03	mannose-6-phosphate isomerase		DW497531	up R	175	N
03 H01	MADS-box protein (AGL84)	Gene Regulation	ES839612	up L	151	N
02 F09	translation initiation factor-like protein		EX172706	up R	230	Y
04 G11	RAN2; GTP binding	Signal Transduction	ES827152	dn R	180	Y
08 H03	Lipoxigenase 3		DT567650	up R	190	Y
03 F02	oligo Peptide transporter, putative	Transport	DW502711	dn R	200	N
06 C03	aquaporin PIP1;3/1;4		DQ402075	up R	466	Y
06E09	aquaporin PIP1;12		GU998829	up L	313	N
				dn R		
08A01	NITRATE TRANSPORTER1/PEPTIDE TRANSPORTER (POT/NAXT) family		na	dn R	300	Y
04D09	Serine carboxypeptidase	Protein Fate	DT456670	dn R	200	Y
01E10	Photosystem I reaction center subunit VI	Photosynthesis	EY197220	up L	189	N
07E12	Cytochrome b6-f complex		ES845215	dn L	285	N
07E02	hydrolase	Cell Growth/Structure	DT465622	dn L	189	N
A5B01	Expansin		DW494258	dn L	278	Y

^a^All reactions were replicated at least twice and two separate gel electrophoresis were performed per reaction.

^b^up and dn represent up-regulation and down-regulation, respectively in root (R) and leaf (L) tissues.

^c^expected size from PCR amplification.

^d^RT-PCR results which correspond to cDNA-AFLP data (Y) and do not (N).

^*^Not available.

### Gene duplication and water deficit stress

The genomic and cytogenetic architecture of tetraploid cotton is a well-known example of gene duplication caused by whole genome duplication and polyploidization [38]. After collectively comparing analyzed TDF sequences, five pairs of duplicated TDFs were identified. As evidenced by different selective primer signatures, fragment sizes, and expression patterns, TDFs from each of five homologous pairs were shown to originate from highly similar but apparently distinct genes (Table 2). Since gene duplication (especially in polyploids) can contribute functional novelty in stress responses, we further analyzed homologous TDFs to have better insights into duplicated gene function under water deficit stress. These TDFs showed similarity to genes coding for proteins such as two hypothetical proteins (for 01E01/02 C11 and A02C03/A04H02), a mannose 6 phosphate isomerase (M6PI) enzyme (for 05 C11/08D03), a plasma membrane intrinsic protein 1 (PIP1) (for 06 C03/06E09), and a superoxide dismutase (SOD) copper chaperone (for A04A08/A04G04).

**Table 2:** Homologous TDFs with high sequence similarity

	**Hypothetical 1**		**Hypothetical 2**		**M6PI^a^**		**PIP1^b^**		**SOD CC^c^**	
	01E01	02 C11	A02C03	A04H02	05 C11	08D03	06 C03	06E09	A04A08	A04G04
MseI/TaqI^*^	AC/TC	TC/TC	TG/CA	CT/CA	GT/AG	GT/AC	GT/AG	TG/AG	TC/CA	AC/CA
bp^%^	223	223	206	342	221	215	537	274	334	310
Expression^†^	dn L	dn R	dn R	up R	up R	up R	up R	up L	up R	dn L
	dn R							dn R		
Similarity to AD genomes^§^	100% A	95% A	98% Gh	99% Gh	98% Gh	99% Gh	99% A	99% A		
	98% D	97% D					98% D	98% D	100% Gh	100% Gh
	100% Gh	98% Gh					99% Gh	99% Gh	100% Gb	100% Gb
	100% Gb	98% Gb					99% Gb	99% Gb		

^a^Mannose 6 phosphate isomerase; ^b^plasma membrane intrinsic protein 1; ^c^superoxide dismutase copper chaperone.

^*^Two nucleotide extensions at 3’-termini of each selective primer set.

^%^Sizes of cDNA-AFLP bands.

^†^up and dn represent up-regulation and down-regulation, respectively in root (R) and leaf (L) tissues.

^§^Genomes from *Gossypium arboreum* (A), *Gossypium raimondii* (D), *Gossypium hirsutum* (Gh), and *Gossypium barbadense* (Gb).

Among them, PIP1-related TDFs (99% identity with each other) belonged to a multi-gene aquaporin water channel membrane protein family in cotton [39]. These TDFs showed very high sequence similarity to GhPIP1;3, GhPIP1;4, GhPIP1;12 and GhPIP1;13 (E value < 9E-174 for 06 C03; < 6E-123 for 06E09 as shown by the multiple sequence alignment (Additional file 2)). According to the prediction of functional residues among aquaporin water channel proteins [40,41], two amino acid substitutions (both Val-Ile conversions) between two TDFs do not affect functional properties. Moreover, these two non-synonymous substitutions belonged to the 4^th^ conserved transmembrane domain, (V/I)GTF(V/I), that is found frequently in cotton EST sequences encoding the aquaporin PIP1 subgroup [39,42]. This points to the importance of either the Val or Ile at these two sites. Our differential expression revealed that an aquaporin TDF, 06 C03 is up-regulated in root by water deficit stress while a homologous TDF, 06E09 is up-regulated in leaf tissue and down-regulated in root tissue. This finding indicates the possible functional, tissue-specific diversification of duplicated PIP1 genes under water deficit stress. Duplicated TDFs from three other homologous pairs also showed differential expression patterns upon water deficit stress, whereas duplicated TDFs of M6PI showed the same pattern of expression (Table 2). These data indicate the possibility that products of duplicated genes might play roles in a tissue dependent manner and that regulatory elements controlling the expression of duplicated genes evolved along with polyploidization.

## Discussion

### Molecular framework under water deficit

Considering that drought-resistant cultivars are not readily available for commercial cotton production, more effort is required to identify or generate traits to withstand soil water deficit stress. In cotton production systems, rain-free periods occurring during reproductive growth are especially damaging. In this study, our objective was to understand a molecular architecture of water deficit stress at the level of gene expression by analyzing differentially expressed transcripts in the field during reproductive growth. Therefore, differentially expressed TDFs identified here can be considered genes functionally active (in case of up-regulation) or inactive (in case of down-regulation) at the stage of adaptation to naturally occurring water deficit stress. Our study revealed more than 500 transcripts with altered gene expression levels. The number of down-regulated genes was 1.5X higher than the number of up-regulated genes. Down-regulated genes occurred more frequently in root tissues. The majority of TDFs with altered expression belonged to functional categories including metabolism, stress/defense, signal transduction, and gene regulation as well as the category unclassified. This result corresponds well with previous findings of other species demonstrating transcriptome alteration under water deficit stress [12,13].

In terms of cotton, a recent microarray expression profiling experiment conducted with plants in a greenhouse reported a total of 2,106 stress responsive-transcripts [31]. The majority of transcripts showed tissue specific expression and a higher number of stress responsive-transcripts were identified in leaf compared to root. In comparison, our study with field grown plants identified fewer stress responsive-transcripts (519), of which, 215 were leaf specific, 293 were root specific, and 11 were expressed in both leaf and root. The candidate gene targets identified in both studies require future work to determine their potential application to improve cotton water use.

Our expression data suggests the involvement of the ROS related defensive pathway since we identified 10 TDFs related to anti-oxidation mechanisms. The increased level of ROS, driven by water deficit stress, can affect cellular components that are oxidized partially or severely [43,44]. Therefore, it is critical that plants protect themselves from harmful oxidations with detoxifying mechanisms by using antioxidants and scavenging agents [45]. DST (Drought and Salt Tolerance), a previously unknown zinc finger protein, was found as a negative regulator of drought and salt stress by repressing H_2_O_2_ accumulation and stomatal closure in an abscisic acid (ABA)-independent manner [44]. In addition, recent studies on developmental and stress-induced cellular processes suggest that ROS and callose deposition are co-regulated, thereby controlling the cell wall matrix adjacent to the plasmodesmata for intercellular redox signal transduction [46]. In our study, one TDF (17-1A09) encoding a callose synthase-like family protein was isolated as a down-regulated gene and many ROS-related TDFs showed up regulation (seven TDFs) or down-regulation (three TDFs). It is of interest to note that this plasmodesmata-related co-regulation of ROS and callose represents a cellular mechanism in the cotton fiber elongation process leading to water uptake following decreased osmotic potential in elongating fiber cells [47]. These findings highlight a possible interconnection between ROS and callose deposition in the areas adjacent to plasmodesmata that cotton employs in response to water deficit stress.

Previous studies have shown that phosphoethanolamine *N*-methyltransferase, vacuolar invertase, and aldo/keto reductase (as identified in this study as TDFs 07B12, A05D02, and A04A09, respectively) are involved in water deficit stress-related defense mechanisms [48-50]. When silenced, the decrease of phosphoethanolamine *N*-methyltransferase produced not only multiple growth defects but also temperature sensitive male sterility in *Arabidopsis*[49]. The vacuolar invertase was shown to be related to water deficit stress in maize [48]. Aldo/keto reductase catalyzes the detoxification reaction of reactive aldehyde groups generated by abiotic stresses thereby providing plants with stress tolerance [50].

It is well known in many plant species that ABA acts as a key hormone in the abiotic plant stress response [51,52]. Upon water deficit stress, stomata in leaves are closed and prevent water loss through transpiration. This process is believed to be regulated by ABA [53,54]. Interestingly, we did not identify any gene related to biosynthesis or action mechanisms of ABA nor genes involved in the regulation of stomata [55]. However, besides the function of ABA in stomatal closure, it was recently shown in *Arabidopsis* and tobacco that interactions possibly occur between water deficit-responsive proteins and HSP chaperones [56,57]. Under water deficit conditions, both HSP70 and HSP90 chaperones were recruited to control stomatal closure, thereby serving as machinery important for stomatal gating. The finding of HSP-coding TDFs also suggests the existence of the HSP related machinery in water deficit stress in cotton. In our study, five HSP-coding TDFs were up-regulated while only one showed down-regulation. Previously, a drought-related alpha-crystalline HSP was identified by differential screening from 10-d drought-stressed *G. arboreum* cotton seedlings and was effective in providing tetraploid cotton plants with drought stress tolerance when over-expressed [58,59]. The potential involvement of HSP-related drought tolerance was also suggested recently in a microarray transcriptome analysis in drought-stressed cotton plants [31].

In accordance with transpiration, molecular mechanisms underlying water uptake and transport are pivotal throughout the plant body. There is accumulating evidence that addresses the relationship between aquaporins and transport of water in various physiological conditions including water deficit stress [60-64]. Aquaporins are frequently identified as water deficit stress responsive genes in diverse plant species [65-67]. In roots of drought stressed chickpea plants, members of the aquaporin gene family appeared up- and down-regulated suggesting that a complex regulation of water status governs plant growth and development through aquaporin activity under water deficit [68]. The identification of two homologous TDFs (06 C03 and 06E09) with similarity to members of the aquaporin water-channel protein family in this study (Table 2 and Additional file 1) indicate the possible involvement of cotton aquaporins under water deficit stressed leaf and root tissues. Recently, the identification of the large cotton aquaporin family illustrated their diverse function [39]. In addition to their fundamental role in intercellular transport of water molecules across the plant body, reports have shown the significance of aquaporins in facilitating leaf CO_2_ conductivity relevant to plant photosynthetic capacity [69-71]. However, the function of aquaporins in water deficit stress tolerance remains unclear as aquaporin genes have not been identified in water deficit stress QTL studies to date [72]. Therefore, more supportive and quantitative data need to follow.

After sequencing 366 excised differentially expressed fragments, we functionally annotated 147 TDFs following sequence verification. Of the 147, we described a number of water deficit stress-responsive genes functionally relevant to metabolism, signal transduction, gene regulation, and stress/defense mechanisms in root and leaf tissues (Figure 3). In addition, homology searches using BlastX did not classify 24.5% of TDFs due to lack of sequence similarity to known proteins. Among those, twenty-seven unclassified TDFs were differentially expressed in root tissue. The abundance of unclassified TDFs identified in this study provide additional transcriptome coverage not represented in EST populations commonly used in microarray experiments. It was reported that unknown genes such as proteins with obscure functions (POF) cover more than 20% of each new genome sequenced with many being species specific [73].

In maize seedlings with water shortage, 5 – 11% of genes were differentially expressed across an array of genetically diverse inbred lines. Also, while many of the genes were not repeatedly identified in different maize lines, more than 40% of the cellular pathways were shared across all the lines examined [74]. In our study, a similar percentage of genes (13%) showed expression level changes upon water deficit stress. Since the genotype in this study is believed to be drought tolerant, it would be interesting to determine if biological pathways highlighted in this study (for example, ROS-, or HSP-related defense mechanisms) would appear in common across an array of cotton genotypes. Transcripts involving the HSP-containing functional group were also identified in a microarray based, water deficit stress response gene expression study using another cotton cultivar, FiberMax 989 [31]. The few water deficit stress-related cotton genes identified in the current study could be used in candidate gene-focused association mapping approaches to identify QTL under drought stress. This approach was previously shown to identify SNPs associated with ABA and sugar levels under water deficit [75].

### Implications of gene duplication in cotton abiotic stress response

As evidenced by recent studies, it is becoming clearer that gene duplication has contributed to adaptive evolution and plant diversification that can lead to evolutionary novelty [76-78]. In our study, four pairs of duplicated genes including two PIP1 homologues were regulated spatially under water deficit stress (Table 2). The differential expression of two duplicated PIP1s did not appear to result from differences of deduced amino acid sequences (Additional file 2). Hence, it is of interest to elucidate factors that lead to the differential response of duplicated genes against stress. These responses can be controlled genetically, epigenetically, or by gene specific *cis*- or *trans*-elements [79]. Recent studies on genetic and epigenetic responses of plant genomes under environmental stimuli suggest that novel stress-induced genotypes such as methylation-sensitive polymorphisms can contribute to crop diversity that may lead to improvements in productivity [80]. In rice, drought stress-induced changes in genome-wide methylation patterns were evaluated using a methylation sensitive cDNA-AFLP method. This revealed a 12.1% site-specific methylation difference between a drought tolerant line (DK151) and its sensitive progenitor line (IR64) when stressed and showed methylation status was regulated tissue specifically [81]. Thus, water deficit responsive molecular changes presented in our study implicate important mechanisms of each gene or coordinated regulation of genes that should be targets of future study.

## Conclusions

In response to field applied water deficit stress, we identified 519 differentially expressed TDFs. This global expression analysis generated sequence information for 147 TDFs and provided gene ontology, distribution of functional categories, and homologous genes. In accordance with data provided here, we highlight possible mechanisms by which cotton responds to water deficit stress. The combination of molecular changes in gene regulation and signal transduction results in increased activity of ROS-related defense mechanisms and HSP-driven protection machinery to provide cellular redox homeostasis and stabilization of functionally/structurally important proteins. A readily available cotton genome sequence will enhance our ability to understand the relationship between gene duplication/polyploidy and the functional, molecular adjustments cotton makes in response to water deficit stress and other environmental stresses.

## Methods

### Plant materials

During 2009, two 8-row plots (6 m row length and 96.5 cm row spacing) of cv. Siokra L-23 were grown at the North Carolina State University Sandhills Research Station near Jackson Springs, NC, USA on a uniformly deep, Candor sand soil with very low water holding capacity. Plots were subjected to irrigated and non-irrigated conditions, respectively, for 4 weeks during reproductive growth. Irrigation was applied supplemental to recorded rainfall weekly using above-ground drip irrigation as described by Campbell and Bauer [82]. Siokra L-23 is generally known to be tolerant to water deficit stress in molecular and physiological levels [83,84]. All plant measurements and tissue samples were obtained mid-day during the flowering period. On each sample date, to confirm differential plant water status, the water potential of uppermost fully expanded leaves was measured on three representative plants from each plot with a pressure bomb (Model 600, PMS Instrument Company, Albany, OR). From the same plants, leaf and root tissues were harvested and immediately frozen in liquid nitrogen for RNA isolation.

### RNA isolation, cDNA synthesis, and cDNA-AFLP

Tissues collected from a single sample date with the greatest water potential difference between well-watered and water deficit stressed plants were used for all nucleic acid analyses. Average water potential of leaf tissue from well-watered and water deficit stressed plants was −1.47 ± 0.13 MPa and −2.58 ± 0.34 MPa, respectively. Total RNA was isolated using the XT buffer system with the addition of chloroform/iso-amyl alcohol extraction and LiCl precipitation steps [85]. Isolation of total RNA and mRNA purification is described previously [39].

A cDNA template was synthesized as follows. First, poly(A) RNA was purified from 200 μg of total RNA using a microPoly(A) Purist Kit (Ambion). After the treatment of DNase, 1 μg of purified mRNA was used to generate first strand cDNA following the manufacturer’s protocol (SuperScript III 1^st^ strand cDNA synthesis kit, Invitrogen) with no RNase H treatment. Double-stranded cDNA was synthesized using an *E.coli* DNA polymerase I (NEB) with RNase H (Invitrogen) at 16°C for 1 hour followed by an additional reaction for 1 hour at 22°C with T4 DNA ligase (NEB) treatment. After inactivation and clean-up, double-stranded cDNA template was digested with *Taq*I and *Mse*I restriction endonucleases sequentially, ligated with two adaptors, and amplified with pre-amplification primer mix (*Mse*I primer: 5’-GATGAGTCCTGAGTAA-3’, *Taq*I primer: 5’-GTAGACTGCGTACCGA-3’). After dilution (1:150) of pre-amplified DNA samples, selective amplification steps were performed with 64 combinations of eight *Mse*I selective primers (5’-GATGAGTCCTGAGTAA*NN’*-3’) and eight IRDye 700-labeled *Taq*I selective primers (5’-/5IRD700/GTAGACTGCGTACCGA*NN’*-3’) for all four templates as indicated in the LI-COR cDNA-AFLP protocol. More detailed cDNA-AFLP procedures are described elsewhere [19,86]. Two separate denaturing polyacryamide gel electrophoresis (PAGE) gels were analyzed for each sample using a LI-COR 4300 DNA Analyzer; one for the preliminary differential expression profiling and the other for gel scanning and band excision. Gel scan images were produced using a LI-COR Odyssey Infrared Imaging System that can detect amplified fragments labeled with IRDye 700. Band excision and recovery of fragments were performed as recommended in the LI-COR manual. To verify technical reproducibility of the cDNA-AFLP reactions, pre-and selective amplification reactions were performed twice independently using three randomly chosen cDNA-AFLP primer sets and the image resolved on a 6.5% PAGE is provided in Additional file 3.

### Cloning, sequencing, and sequence analysis

After recovery of individual TDFs from cDNA-AFLP, fragments were re-amplified with primers used for pre-amplification and subcloned into pCR-TOPO cloning vector systems (Invitrogen) following clean-up of PCR reaction products with a Wizard PCR clean-up kit (Promega). Three to four colonies from each band were bi-directionally sequenced. Details about cloning, sequencing, and RT-PCR amplification procedures have been described previously [39]. Sequence information of gene-specific primers used for RT-PCR is available upon request. For the identity of sequenced TDFs, homology-based blast searches from the National Center for Biotechnology Information (NCBI, http://www.ncbi.nlm.nih.gov/) were performed, and predicted protein sequences and ESTs with highest similarity were annotated. Functional annotation of each TDF was also performed following the Gene Ontology tool [37]. When necessary, cotton assembly contig sequences were used for additional blast homology searches with a cotton46 version that is available from the Comparative Evolutionary Genomics of Cotton web site (http://cottonevolution.info/). Alignment and assembly of sequences were processed using VectorNTI (Invitrogen) and ClustalW2 programs (http://www.ebi.ac.uk/Tools/msa/clustalw2/).

## Abbreviations

AFLP = Amplified fragment length polymorphism ; RT-PCR = Reverse transcription-polymerase chain reaction ; TDF = Transcript derived fragment; ROS = Reactive oxygen species ; HSP = Heat shock protein.

## Authors’ contributions

WP participated in the experimental design, performed experiments, analyzed data, and wrote the manuscript. BES performed DNA sequencing and participated in writing the manuscript. PJB participated in the experimental design and writing the manuscript. BTC participated in the experimental design, supervised all procedures, analyzed data and wrote the manuscript. All authors read and approved the final manuscript.

## Supplementary Material

Additional file 1 Annotation of 147 differntially expressed TDFs. Click here for file

Additional file 2 **Clustal W2 multiple sequence alignment of two TDF homologs and cotton aquaporins.** Priming sites for two selective markers are shown above alignment panels. Three different nucleotide sequences are highlighted and predicted amino acid sequences are shown below the alignment. Only coding regions are used for comparisons and one Taq-GT selective marker site for the 06 C03 is not shown here. Numbers to the right represent nucleotide positions from ATG of ESTs and from the end of TDFs, respectively. Click here for file

Additional file 3 **cDNA-AFLP gel image with three primer sets for two technical replicates.** Two independent reactions were performed and loaded side by side to show reproducible amplification. wL and dL denote samples from irrigated and water deficit stressed leaf, respectively. wR and dR denote samples from irrigated and water deficit stressed root, respectively. Asterisk means replicated reaction. TDFs with reproducible differences are shown in green arrows and a red arrow shows a non-reproducible TDF. Size markers are presented in right side. Click here for file
