# Supplementary material for: Genome-wide identification of differentially expressed genes under water deficit stress in upland cotton (Gossypium hirsutum L.)
Source: BMC Plant Biol. 2012 Jun 15;12:90. doi: 10.1186/1471-2229-12-90 (PMC3438127; doi:10.1186/1471-2229-12-90)
Supplement: Additional file 2 — Clustal W2 multiple sequence alignment of two TDF homologs and cotton aquaporins. Priming sites for two selective markers are shown above alignment panels. Three different nucleotide sequences are highlighted and predicted amino acid sequences are shown below the alignment. Only coding regions are used for comparisons and one Taq-GT selective marker site for the 06 C03 is not shown here. Numbers to the right represent nucleotide positions from ATG of ESTs and from the end of TDFs, respectively. [file 1471-2229-12-90-S2.doc]

Additional File 2. Clustal W2 multiple sequence alignment of two TDF homologs and cotton aquaporins. Priming sites for two selective markers are shown above alignment panels. Three different nucleotide sequences are highlighted and predicted amino acid sequences are shown below the alignment. Only coding regions are used for the comparison and one *Taq*-GT selective marker site for the 06C03 is not shown here. Numbers to the right represent nucleotide positions from ATG of ESTs and from the end of TDFs, respectively.

*Mse*I-AG

GhPIP1_12 CTTGGTGCTATCTGTGGAGCTGGTGTAGTGAAAGGAT***TCGA***AGGAGACAGTAGATATGAG 480

GhPIP1_13 CTTGGTGCTATCTGTGGAGCTGGTGTAGTGAAAGGAT***TCGA***AGGAGACAGTAGATATGAG 480

GhPIP1_4 CTTGGTGCTATCTGTGGAGCTGGTGTAGTGAAAGGAT***TCGA***AGGAGACAGTAGATATGAG 480

GhPIP1_15 CTTGGTGCTATCTGTGGAGCTGGTGTAGTGAAAGGAT***TCGA***AGGAGACAGTAGATATGAG 480

GhPIP1_3 CTTGGTGCTATCTGTGGAGCTGGTGTAGTGAAAGGAT***TCGA***AGGAGACAGTAGATATGAG 480

06C03 --------------------------------------***CGA***AGGAGACAGTAGATATGAG 22

06E09 --------------------------------------***CGA***AGGAGACAGTAGATATGAG 22

GhPIP1_12 ATGTTGGGTGGTGGAGCCAATGTTGTGAACCATGGCTACACCAAAGGTGATGGTCTTGGT 540

GhPIP1_13 ATGTTGGGTGGTGGAGCCAATGTTGTGAACCATGGCTACACCAAAGGTGATGGTCTTGGT 540

GhPIP1_4 ATGTTGGGTGGTGGAGCCAATGTTGTGAACCATGGCTACACCAAAGGTGATGGTCTTGGT 540

GhPIP1_15 ATGTTGGGTGGTGGAGCCAATGTTGTGAACCATGGCTACACCAAAGGTGATGGTCTTGGT 540

GhPIP1_3 ATGTTGGGTGGTGGAGCCAATGTTGTGAACCATGGCTACACCAAAGGTGATGGTCTTGGT 540

06C03 ATGTTGGGTGGTGGAGCCAATGTTGTGAACCATGGCTACACCAAAGGTGATGGTCTTGGT 82

06E09 ATGTTGGGTGGTGGAGCCAATGTTGTGAACCATGGCTACACCAAAGGTGATGGTCTTGGT 82

GhPIP1_12 GCTGAAATCGTTGGCACTTTTATTCTTGTCTACACTGTTTTCTCTGCTACTGATGCCAAG 600

GhPIP1_13 GCTGAAATCATTGGCACTTTTGTTCTTGTCTACACTGTTTTCTCTGCTACTGATGCCAAG 600

GhPIP1_4 GCTGAAATCGTTGGCACTTTTATTCTTGTCTACACTGTTTTCTCTGCTACTGATGCCAAG 600

GhPIP1_15 GCTGAAATCGTTGGCACTTTTATTCTTGTCTACACTGTTTTCTCTGCTACTGATGCCAAG 600

GhPIP1_3 GCTGAAATCATTGGCACTTTTGTTCTTGTCTACACTGTTTTCTCTGCTACTGATGCCAAG 600

06C03 GCTGAAATCATTGGCACTTTTGTTCTTGTCTACACTGTTTTCTCTGCTACTGATGCCAAG 142

06E09 GCTGAAATCGTTGGCACTTTTATTCTTGTCTACACTGTTTTCTCTGCTACTGATGCCAAG 142

Val/Ile Val/Ile

GhPIP1_12 AGAAATGCCAGAGACTCTCACGTTCCTATTTTGGCTCCCCTACCTATAGGGTTTGCAGTG 660

GhPIP1_13 AGAAATGCCAGAGACTCTCACGTTCCTATTTTGGCTCCCCTACCTATAGGGTTTGCAGTG 660

GhPIP1_4 AGAAATGCCAGAGACTCTCACGTTCCTATTTTGGCTCCCCTACCTATAGGGTTTGCAGTG 660

GhPIP1_15 AGAAATGCCAGAGACTCTCACGTTCCTATTTTGGCTCCCCTACCTATAGGGTTTGCAGTG 660

GhPIP1_3 AGAAATGCCAGAGACTCTCACGTTCCTATTTTGGCTCCCCTACCTATAGGGTTTGCAGTG 660

06C03 AGAAATGCCAGAGACTCTCACGTTCCTATTTTGGCTCCCCTACCTATAGGGTTTGCAGTG 202

06E09 AGAAATGCCAGAGACTCTCACGTTCCTATTTTGGCTCCCCTACCTATAGGGTTTGCAGTG 202

*Taq*I-TG

GhPIP1_12 TTCTTGGTTCATTTGGCCACCATTCCCATCACTGGAACTGG***TATTAA***CCCAGCAAGGAGT 720

GhPIP1_13 TTCTTGGTTCATTTGGCCACCATTCCCATCACTGGAACTGG***TATTAA***CCCAGCAAGGAGT 720

GhPIP1_4 TTCTTTGTTCATTTGGCCACCATTCCCATCACTGGAACTGG***TATTAA***CCCAGCAAGGAGT 720

GhPIP1_15 TTCTTGGTTCATTTGGCCACCATTCCCATCACTGGAACTGG***TATTAA***CCCAGCAAGGAGT 720

GhPIP1_3 TTCTTGGTTCATTTGGCCACCATTCCCATCACTGGAACTGG***TATTAA***CCCAGCAAGGAGT 720

06C03 TTCTTGGTTCATTTGGCCACCATTCCCATCACTGGAACTGG***TATTAA***CCCAGCAAGGAGT 262

06E09 TTCTTGGTTCATTTGGCCACCATTCCCATCACTGGAACTGG***CATTA***-------------- 248

Gly

GhPIP1_12 CTTGGAGCTGCCATTATATATAACAAAGACCATGCATGGGATGATCATTGGATCTTCTGG 780

GhPIP1_13 CTTGGAGCTGCCATTATATATAACAAAGACCATGCATGGGATGATCATTGGATTTTCTGG 780

GhPIP1_4 CTTGGAGCTGCCATTATATATAACAAAGACCATGCATGGGATGATCATTGGATCTTCTGG 780

GhPIP1_15 CTTGGAGCTGCCATTATATATAACAAAGACCATGCATGGGATGATCATTGGATTTTCTGG 780

GhPIP1_3 CTTGGAGCTGCCATTATATATAACAAAGACCATGCATGGGATGATCATTGGATTTTCTGG 780

06C03 CTTGGAGCTGCCATTATATATAACAAAGACCATGCATGGGATGATCATTGGATCTTCTGG 322

GhPIP1_12 GTTGGGCCCCTTCATTGGACC--------------------------------------- 801

GhPIP1_13 -TTGGGCCCCTTCATTGGACC--------------------------------------- 800

GhPIP1_4 GTTGGACCC-TTCATTGGAGCTGCTCTTGCTGCAATTTACCACCAGATAATCATCAGGGG 839

GhPIP1_15 GTTGGACCC-TTCATTGGAGCTGCTCTTGCTGCAATTTACCACCAGATAATCATCAGGGC 839

GhPIP1_3 GTTGGACCC-TTCATTGGAGCTGCTCTTGCTGCAATTTACCACCAGATAATCATCAGGGC 839

06C03 GTTGGACCC-TTCATTGGAGCTGCTCTTGCTGCAATTTACCACCAGATAATCATCAGGGC 381

STOP

GhPIP1_4 CATTCCTTTCAAGACCAGAGCT***TGA*** 864

GhPIP1_15 CATTCCTTTCAAGACCAGAGCT***TGA*** 864

GhPIP1_3 CATTCCTTTCAAGACCAGAGCT***TGA*** 864

06C03 CATTCCTTTCAAGACCAGAGCT***TGA*** 406
