# Supplementary material for: Genome-wide identification of differentially expressed genes under water deficit stress in upland cotton (Gossypium hirsutum L.)
Source: BMC Plant Biol. 2012 Jun 15;12:90. doi: 10.1186/1471-2229-12-90 (PMC3438127; doi:10.1186/1471-2229-12-90)
Supplement: Additional file 3 — cDNA-AFLP gel image with three primer sets for two technical replicates. Two independent reactions were performed and loaded side by side to show reproducible amplification. wL and dL denote samples from irrigated and water deficit stressed leaf, respectively. wR and dR denote samples from irrigated and water deficit stressed root, respectively. Asterisk means replicated reaction. TDFs with reproducible differences are shown in green arrows and a red arrow shows a non-reproducible TDF. Size markers are presented in right side. [file 1471-2229-12-90-S3.ppt]

## Slide 1
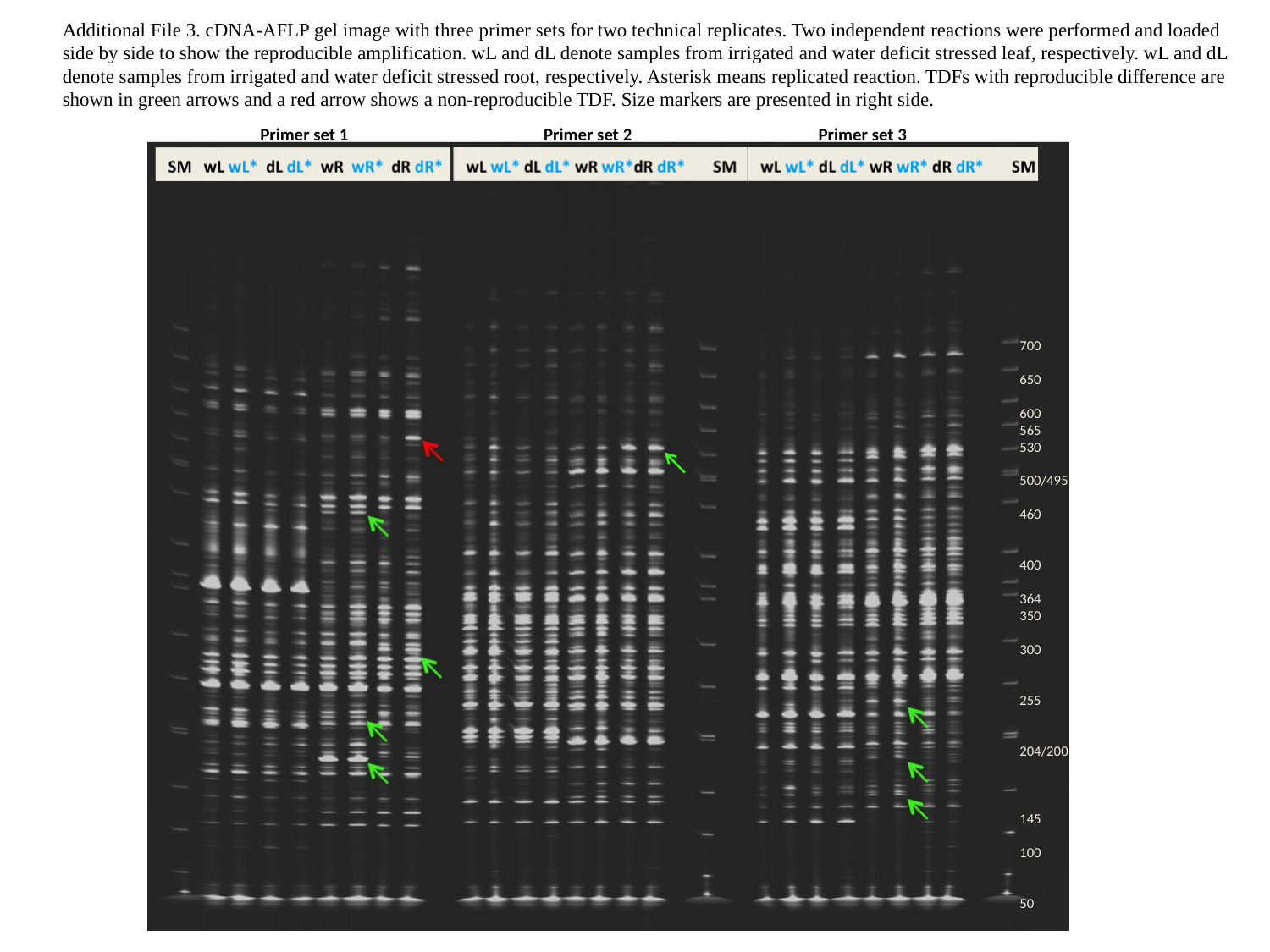

Additional File 3. cDNA-AFLP gel image with three primer sets for two technical replicates. Two independent reactions were performed and loaded side by side to show the reproducible amplification. wL and dL denote samples from irrigated and water deficit stressed leaf, respectively. wL and dL denote samples from irrigated and water deficit stressed root, respectively. Asterisk means replicated reaction. TDFs with reproducible difference are shown in green arrows and a red arrow shows a non-reproducible TDF. Size markers are presented in right side.
Primer set 1
Primer set 2
Primer set 3
700
650
600
565
530
500/495
460
400
364
350
300
255
204/200
145
100
50
